# Comparison of Kaposi disease outlines in patients with and without HIV infection in two tertiary care hospitals in Bucharest, Romania

**DOI:** 10.1186/1471-2334-14-S7-P20

**Published:** 2014-10-15

**Authors:** Oana Săndulescu, Ioana Ţiu, Raluca Jipa, Anca Streinu-Cercel, Șerban Benea, Olga Simionescu, Adrian Streinu-Cercel, Adriana Hristea

**Affiliations:** 1 Carol Davila University of Medicine and Pharmacy, Bucharest, Romania; 2 National Institute for Infectious Diseases "Prof. Dr. Matei Balş", Bucharest, Romania; 3 First Department of Dermatology, Colentina Clinical Hospital, Bucharest, Romania

## Background

Kaposi disease (KD) displays polymorphic manifestations, ranging from minimal cutaneous involvement to extensive visceral disease. Its clinical outline is different in HIV-positive patients compared to the classical non-HIV-related form that is diagnosed more often in elderly patients without other significant immune impairment.

## Methods

A retrospective study on KD was performed in two academic centers, tertiary-care hospitals with national addressability in Romania. Two groups were comparatively studied: HIV-infected patients diagnosed in the National Institute for Infectious Diseases “Prof.Dr. Matei Balş” (HIV-positive group), and non-HIV patients diagnosed in the first Clinic of Dermatology, Colentina Clinical Hospital (HIV-negative group). The statistical analysis was performed using IBM SPSS Statistics v.22 (Chicago, USA).

## Results

A total number of 71 cases, 30 in the HIV-positive group and 41 in the HIV-negative group were identified. The non-HIV patients were benign European form and immunosuppressed other than HIV, respectively. There was a male predominance, with a male-to-female ratio in HIV-positive and HIV-negative patients of 2:1 and 4.1:1 respectively. The mean age at KD diagnosis was 41.6±15.0 years in HIV-positive and 70.2±11.8 years in HIV-negative patients.

The mean number of comorbidities was 3 in the HIV-positive group compared to 1 in HIV-negative, p=0.011). In the HIV-positive group, 17 patients (56.7%) were classified Mitsuyasu stage 1, 6 (20%) stage 2, 2 (6.7%) stage 3 and 5 (16.7%) stage 4. In the HIV-negative group, all 41 patients were Mitsuyasu stage 1.

In the HIV-positive group all patients received antiretroviral therapy and only 6 (20%) received other types of targeted therapy for KD: topical (5, 16.7%), systemic (5, 16.7% – interferon, etc.), chemotherapy (2, 6.7%), local radiotherapy (2, 6.7%). In the HIV-negative group, all patients received specific treatment, such as: topical (24, 58.5%), systemic (14, 34.1% – dapsone, pentoxifylline), chemotherapy (2, 4.9%), local radiotherapy (7, 17.1%), electrocauterization (15, 36.6%), surgical excision (4, 9.8%).

Loss for follow-up appeared to be less frequent in the HIV-positive group (7 patients, 35%) than in the HIV-negative group (35 patients, 85.4%) but occurred faster in the HIV-positive group, after a mean interval of 8.6±3.4 vs. 15.9±37.3 months in HIV-negative group. Overall survival was 60% (HIV-positive) and 100% (HIV-negative).

## Conclusion

This work has identified two different outlines of KD, an aggressive progression with high mortality in HIV-positive patients, and a gradual progression, with virtually no short-term mortality, in elderly patients. In the HIV-positive group, very few patients received other types of treatment for KD, apart from antiretroviral therapy.

